# Transcriptome analysis reveals molecular anthelmintic effects of procyanidins in *C*. *elegans*

**DOI:** 10.1371/journal.pone.0184656

**Published:** 2017-09-19

**Authors:** Verena Spiegler, Andreas Hensel, Jochen Seggewiß, Milena Lubisch, Eva Liebau

**Affiliations:** 1 Institute of Pharmaceutical Biology and Phytochemistry, University of Münster, Münster, Germany; 2 Institute of Human Genetics, University Hospital Münster, Münster, Germany; 3 Department of Molecular Physiology, Institute for Animal Physiology, Münster, Germany; New England Biolabs Inc, UNITED STATES

## Abstract

Worldwide, more than 1 billion people are affected by infestations with soil-transmitted helminths and also in veterinary medicine helminthiases are a severe threat to livestock due to emerging resistances against the common anthelmintics. Proanthocyanidins have been increasingly investigated for their anthelmintic properties, however, except for an interaction with certain proteins of the nematodes, not much is known about their mode of action. To investigate the anthelmintic activity on a molecular level, a transcriptome analysis was performed in *Caenorhabditis elegans* after treatment with purified and fully characterized oligomeric procyanidins (OPC). The OPCs had previously been obtained from a hydro-ethanolic (1:1) extract from the leaves of *Combretum mucronatum*, a plant which is traditionally used in West Africa for the treatment of helminthiasis, therefore, also the crude extract was included in the study. Significant changes in differential gene expression were observed mainly for proteins related to the intestine, many of which were located extracellularly or within cellular membranes. Among the up-regulated genes, several hitherto undescribed orthologues of structural proteins in humans were identified, but also genes that are potentially involved in the worms’ defense against tannins. For example, T22D1.2, an orthologue of human basic salivary proline-rich protein (PRB) 2, and *numr-1* (nuclear localized metal responsive) were found to be strongly up-regulated. Down-regulated genes were mainly associated with lysosomal activity, glycoside hydrolysis or the worms’ innate immune response. No major differences were found between the groups treated with purified OPCs versus the crude extract. Investigations using GFP reporter gene constructs of T22D1.2 and *numr-1* corroborated the intestine as the predominant site of the anthelmintic activity. The current findings support previous hypotheses of OPCs interacting with intestinal surface proteins and provide the first insights into the nematode’s response to OPCs on a molecular level as a base for the identification of future drug targets.

## Introduction

Worldwide, more than 1.5 billion people are affected by infestations with intestinal nematodes [1,2] and also in veterinary medicine, helminths pose a severe threat to livestock [3–5]. Commonly used effective drugs against these diseases include the benzimidazoles, pyrantel pamoate, ivermectin or levamisole [6–8], however, their number is limited and reports about emerging resistances are dramatic, especially in veterinary medicine [9,10]. Polyphenols, particularly condensed tannins, are a class of natural compounds that have recently been extensively investigated *in vitro* and *in vivo* for their anthelmintic activities (for reviews see [11–18]). Condensed and hydrolysable tannins have been reported to cause a variety of detrimental effects *in vitro* on different nematode species at different life stages. These include the inhibition of egg hatch, larval exsheathment, larval migration, feeding and larval development as well as lethal effects in adults (for review see [12,14,18]). Regarding their mode of action, tannins are known for their astringent properties and likely interact with certain proteins, especially those rich in proline or hydroxyproline, mainly via hydrogen bonding or hydrophobic interaction [19–25]. Several microscopic investigations have indicated that in particular the nematode cuticle, the buccal cavity and the intestine provided potential binding sites for tannins [12,26–34]. Also flavonoids, another major subgroup of polyphenols, exert direct anthelmintic effects in some cases [35–39] and no effect in others [33], depending on the exact flavonoid structure and the test system used [40–43]. Moreover, flavonoids have been shown to enhance the activity of tannins in a synergistic manner [38]. However, despite the intensive research in this field, there are hardly any investigations concerning the anthelmintic mode of action of polyphenolic compounds on a molecular level. Insights into the molecular mechanism would not only contribute to a better understanding of the anthelmintic effect of polyphenols, but might also lead to potential new and innovative targets for the development of novel anthelmintic drugs in the future. Therefore, the current study aims at investigating the molecular effects of a polyphenol rich extract and isolated procyanidins from the leaves of *Combretum mucronatum*, a plant traditionally used in West Africa for the treatment of intestinal helminthiasis [44], by transcriptome analysis using the model nematode *Caenorhabditis elegans*.

## Materials and methods

### Plant material and chemicals

Leaves from *C*. *mucronatum* were harvested in the Bosomtwi-Atwima-Kwanwoma area in the Ashanti region of Ghana, located between 0.15–2.25°W and 5.50–7.46°N (Campus of the Kwame Nkrumah University of Science and Technology, Kumasi), by a university technician between April and May 2011. *C*. *mucronatum* is a plant species widely distributed within West Africa. The plant is not endangered and not protected according to relevant international listings. For collection of the plant material no specific permission was required. The plant material was air dried for two weeks at room temperature after botanical identification and reference samples were stored at the Institute for Pharmaceutical Biology and Phytochemistry, Münster (voucher no. IPBP-324). If not stated otherwise, all chemicals were purchased from VWR (Darmstadt, Germany).

### Preparation of plant extracts and fractions

A hydroethanolic extract (1:1 v/v) was prepared in a drug-solvent ratio of 1:10 as described previously [45]. By bioassay-guided fractionation a fraction was obtained that entirely consisted of oligomeric procyanidins composed of epicatechin building blocks and which has been characterized previously [45]. Briefly, the crude extract was partitioned between ethyl acetate and water and a methanol soluble fraction of the aqueous partition was subsequently fractionated by MPLC on an RP-18 stationary phase (RP-18, 18–32 μm, 100 Å, 36 × 500 mm (BESTA Technik, Wilhelmsfeld, Germany), flow 4 mL/min, step gradient MeOH 10% (50 min) → MeOH 30% (2 h) → MeOH 50% (2 h) → MeOH (2 h), fraction size 16 mL). Subfraction “H3” (992–1152 mL, yield: 1.29 g) was used in the experiments. The anthelmintic activity of extract and fractions has been assessed using *C*. *elegans* as described in [45].

### Culture and treatment of *C*. *elegans*

Cultures of *C*. *elegans* wildtype (N2 Bristol strain) obtained from the Caenorhabditis Genetics Center, University of Minnesota, were maintained as described by [46] and grown at 20°C on petri dishes containing Nematode Growth Medium (NGM) supplemented with 800 μL of *Escherichia coli* OP50 strain as a food source [46]. Age synchronous cultures were obtained by alkaline bleaching (600 μL NaOCl solution (Sigma-Aldrich, Germany), 100 μL 10 M NaOH solution, 1300 μL H_2_O) for 7 minutes. Eggs were allowed to hatch overnight in M9 buffer [47], L1 larvae were transferred to fresh NGM plates seeded with *E*. *coli* OP50 and incubated at 20°C until the development to young adults. Test solutions were prepared in concentrations of 2 mg/mL, corresponding to the LC_10_ after 6 h of incubation, and its dilutions to 0.2 mg/mL and 0.02 mg/mL of fraction H3 and 0.2 mg/mL of the EtOH-H_2_O (1:1) extract in 10 mL M9. 100 μL DMSO were used as a solubilizer, therefore a solution of 1% DMSO was used as a negative control. The solutions were centrifuged for 1 min at 2000 × *g* and the supernatant was poured into a 75 cm^2^ tissue culture flask. Treatment of the worms for the qPCR experiment was performed accordingly, using 4 mL of test solution in a 25 cm^2^ tissue culture flask. Prior to treatment, worms were rinsed off the NGM plates and the worm suspension was washed three times with M9 buffer to remove remaining bacteria. Approximately 500 to 1000 worms were transferred to each tissue culture flask, and incubated for 6 h at 20° C. After the incubation time, worms were poured into a fresh 15 mL Falcon tube for centrifugation at 1500 × *g* for 2 min. The supernatant was removed, worms were washed once in M9, transferred to a 1.5 mL reaction tube and after removal of the supernatant, the tube containing the worm pellet was snap frozen in liquid nitrogen. The procedure was repeated twice to obtain three independent replicates. Both, the microarray as well as the qPCR experiments were conducted with three biological replicates per sample.

### RNA isolation

RNA was isolated using the RNeasy^®^ Mini Kit (Qiagen, Germany). In the first step, lysis of the worms was achieved by grinding the pellet in a total of 600 μL lysis buffer under repeated freezing in liquid nitrogen. Further isolation, including an on-column DNAse I digest (Qiagen, Germany), was performed according to the manufacturer’s instructions. For the qPCR samples, the on-column DNAse I digest was replaced by digestion of the eluted RNA using the Invitrogen™ Ambion™ TURBO DNA-*free* Kit (Thermo Scientific, USA) according to the manufacturer’s instructions.

### Transcriptome analysis

RNA concentration and purity were measured using a Nanodrop spectrophotometer (Thermo Fisher Scientific Inc., Waltham, MA, USA). RNA integrity was checked using an Agilent Bioanalyzer (software version B.02.07.SI532) on a RNA Nano Chip (both Agilent Technologies Inc., Santa Clara, CA, USA). Labelling and hybridization of the RNA were performed as described in the manufacturer’s Affymetrix GeneChip^®^ WT PLUS Reagent Kit. Fragmented and labeled library was hybridized to an Affymetrix Gene 1.0 ST *C*. *elegans* Array containing 28,305 genes and a total of 638,442 probes (24 probes per gene). Following hybridization, the arrays were washed and stained using the Affymetrix GeneChip Fluidics Station 450 and scanned using the Affymetrix GeneChip Scanner 3000 7G. After scanning, first quality analyses were made using the Affymetrix Expression Console (Affymetrix) and Partek Genomics Suite software (Partek Inc., St. Louis, MO, USA) to check the spike-in controls. Microarray data quality was checked as recommended by the manufacturer and by the quality metrics in the Partek Genomics Suite software (Partek Inc., St. Louis, MO, USA). Statistical analyses of microarray data were performed using the Partek Genomics Suite. CEL-files (containing raw expression measurements) were imported to Partek GS. The robust multi-array average (RMA) algorithm was used for normalization. The array data were quantile-normalized and log-2 transformed. For each probe, a one-way analysis of variance was performed: Y_ij_ = μ + Group_i_ + ε_ij_, where Y_ij_ represents the j^th^ observation on the i^th^ Group and μ is the common effect for the whole experiment. ε_ij_ represents the random error present in the j^th^ observation on the i^th^ Group. The errors ε_ij_ are assumed to be normally and independently distributed with mean 0 and standard deviation δ for all measurements [48]. For each probe, Fisher's least significant difference was tested to statistically compare the difference between the means of the groups´ expression measurements. A false-discovery-rate of ≤ 0.05 was used as a threshold for significance [49]. Only genes with a fold-change of ± 2.0 were regarded as significantly changed in expression. Raw data are available from Gene Expression Omnibus (accession number: GSE101680), processed data are given as supplementary file (S1 Table).

Gene ontology (GO) enrichment analysis and functional classification of genes was performed using the Database for Annotation, Visualization and Integrated Discovery (DAVID; 6.8 Beta) [50,51].

WormBase (wormbase.org) was referred to regarding information about single genes and corresponding protein sequences.

### qPCR

First strand cDNA was synthesized from 1 μg of the digested RNA samples using oligo (dT) primers according to the manufacturer’s instructions (Transcriptor First Strand cDNA Synthesis Kit; Roche Applied Science, Germany). The relative gene expression was quantified for two of the most down-regulated genes, *pud-1*.*1* and *ugt-44*, and for two of the most up-regulated genes, T22D1.2 and F13E9.4. Primers were designed using the open software PerlPrimer v1.1.20 [52], double checked using the online tool Primer 3 v. 0.4.0 [53,54] and purchased from Eurofins Genomics (Ebersberg, Germany). *pmp-3* was used as the housekeeping gene and primer sequences were taken from [55]. A list of all primer sequences is given in Table 1.

**Table 1:** Overview of primers used for qPCR.

Gene	Sequence name	Direction	Sequence (5’ -> 3’)
*ugt-44*	F01D4.2	fwd	TATAATGTGACCCTTCTGTTGC
		rev	TTCCCGAGTTCATAACCCA
*pud-1*.*1*	F15E11.13	fwd	ATGTTGTGATGTTCCAGCC
		rev	CCTTTGTTATCCACTTCAGTTCTC
T22D1.2	T22D1.2	fwd	GGTAATGCTTCTCGTCGTCC
		rev	TCTGCAGAAAGATCCTGTGGT
F13E9.4	F13E9.4	fwd	AGTGTTCAAGCCCAATACATCC
		rev	CTTGTGTTTGACTGAATTCCCT
pmp-3*	C54G10.3	fwd	GTTCCCGTGTTCATCACTCAT
		rev	ACACCGTCGAGAAGCTGTAGA

*pmp-3 was used as the reference gene, its primer sequences were taken from [55].

Fwd: forward, rev: reverse.

RT-PCR was performed in a CFX96 Real-Time System (BioRad, Germany) using the iTaq™ Universal SYBR® Green Supermix (BioRad, Germany) and Hard-Shell® 96-Well PCR Plates (BioRad, Germany). Each well contained a total volume of 20 μL, including 1 μL forward and reverse primers at a final concentration of 400 nmol/L and 2 μL DNA template containing 10 ng cDNA or 2 μL of the respective -RT control. All samples were quantified in duplicate, -RT controls were applied once for every gene and sample. Additionally, no template controls containing 2 μL nuclease free H_2_O instead of the DNA template were applied once per gene. Cycling conditions were set as follows: Initial denaturation for 30 s at 95° C, followed by 40 cycles of 5 s at 95° C and 30 s at 60° C. After further 31 s at 65° C, the temperature was increased for 60 cycles by 0.5° C per 5 s cycle for melting curve analysis. The quantitative gene expression of treated versus untreated *C*. *elegans* was evaluated with the CFX Manager^TM^ software (Bio-Rad, Germany) based on the comparative C_T_ method and levels of gene expression were compared to the samples of the negative control (1% DMSO).

### GFP reporter gene construct and transgenic *C*. *elegans*

#### pT22D1.2+SP::GFP

A fragment of 2000 bp of T22D1.2 including the promotor region and the signal peptide (SP) which was determined using the online tool SignalP 4.1 [56] was amplified from *C*. *elegans* genomic DNA using the Finnzymes Phusion® High-Fidelity DNA Polymerase Kit (Thermo Scientific, USA). 95T22D1.2pSPBamS was used as forward primer (5’-CGACTCTAGAGGATCCTCTTTTT-TTACCAGAATGTTCA-3’), 95T22D1.2pSPKpnA (5’-TCATTTTTTCTACCGGTACCGGAGAA-GCAGTCTCACCAACT-3’) was used as reverse primer. The amplicon was cloned into pPD95.77, which is part of the Fire Lab *C*. *elegans* Vector Kit and was kindly provided by A. Fire. A solution of the pT22D1.2::GFP construct (80 μg/mL) and the pRF4 plasmid encoding *rol-6* (50 μg/mL) as a marker gene were co-injected into the germline of young adult *C*. *elegans* [57,58].

#### pT22D1.2::GFP

A fragment of 2000 bp including only the promotor region of T22D1.2 was amplified from *C*. *elegans* genomic DNA as described above using primers 95T22D1.2pSPBamS (forward; 5’-CGACTCTAGAGGATCC TCTTTTTTTACCAGAATGTTCA-3’) and 95T22D1.2PrKpnAS (reverse; 5’-TCATTTTTTCTACCGGTACCTGTGAATTCAATGAGTGATAC-3’). Strain JF88 mtEx63 [numr-1p::numr-1::GFP + rol-6(su1006)] was obtained from the Caenorhabditis Genetics Center at the University of Minnesota. The transgenic strains were treated as follows: Approximately 10 worms per well were incubated for 6 h in a 24-well-microtiter plate containing 500 μL of the crude hydro-ethanolic extract at concentrations of 0.2 mg/mL and 2 mg/mL or the negative control (1% DMSO in M9 buffer). Each treatment was performed in four replicates. GFP expression was analysed by fluorescence microscopy (Zeiss LSM 510, Zeiss, Germany).

## Results

### Transcriptome analysis

**Fig 1:**
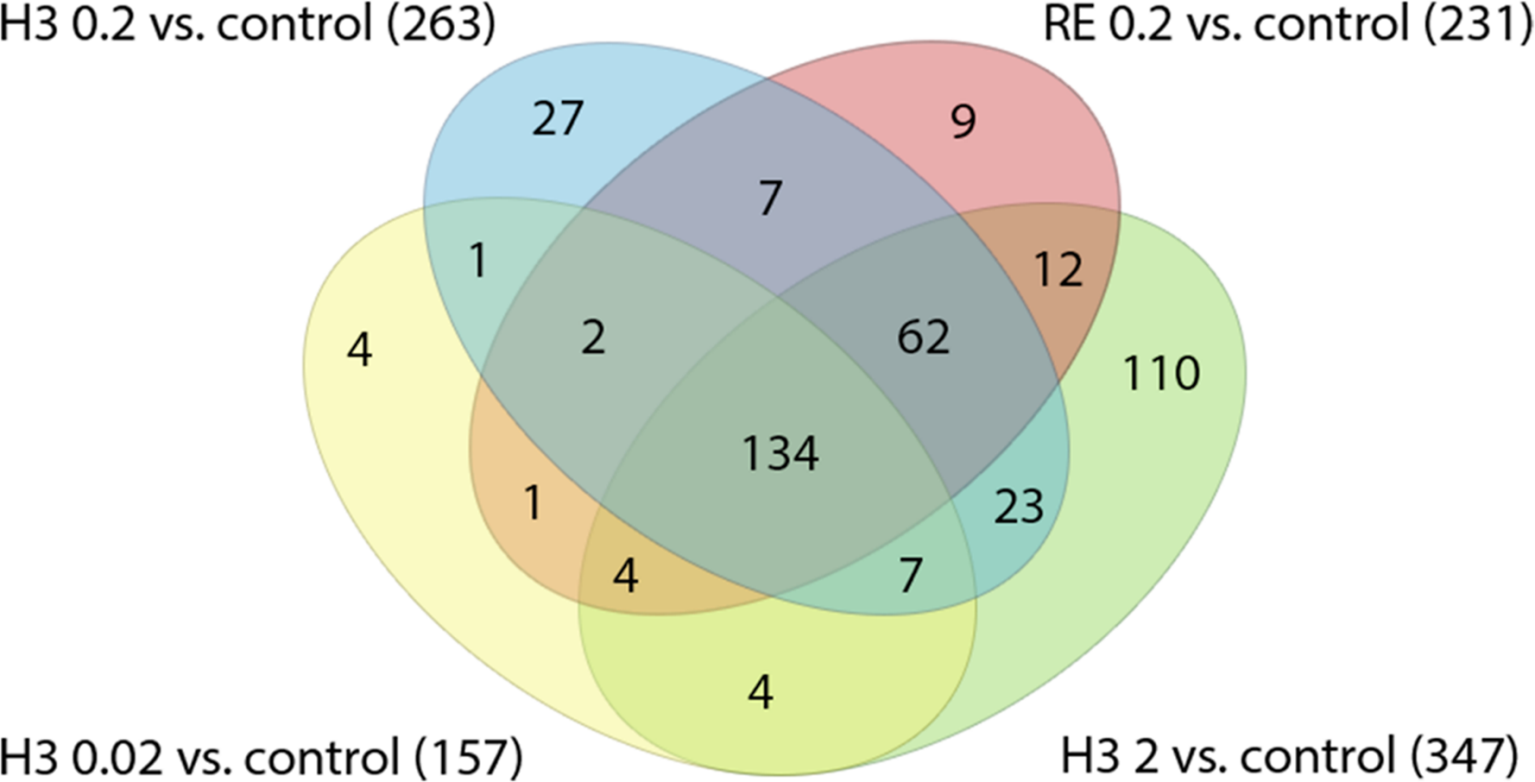
Venn diagram showing the number of differentially regulated genes per treatment vs. the untreated negative control (DMSO 1%). H3: OPC enriched fraction “H3”; RE: crude extract (ethanol: water 1:1). 0.02, 0.2 and 2: concentrations of 0.02 mg/mL, 0.2 mg/mL and 2 mg/mL resp., per treatment. Numbers in brackets indicate the number of differentially regulated genes per group.

From the leaves of *Combretum mucronatum*, a herbal remedy which is widely used as anthelmintic remedy in West Africa [44], an extract was obtained which was strongly enriched with oligomeric procyanidins (OPC). By bioassay-guided fractionation of this extract, fraction H3 was obtained, which contained a mixture of oligomeric procyanidins, which recently have been characterized in detail [45]. All microarray results were conducted with three biological replicates per sample. As shown in Fig 1, treatment with the OPC enriched fractions or the crude hydro-ethanolic extract caused a considerable change in the transcriptome of all treated groups. The number of differentially regulated genes increased with an increase in the concentration of the respective treatment: While “only” 157 genes were up- or down-regulated vs the negative control at 0.02 mg/mL of fraction H3, at 0.2 mg/mL the number increased to 263 and finally to 347 genes at 2 mg/mL. 231 genes were differentially regulated in the group treated with crude extract (RE) at 0.2 mg/mL.

To identify the worm’s key responses to the tannin treatment, further analysis of the data focused on the 134 genes that were differentially expressed in all treatment groups compared to the control (S2 Table). Annotation clustering was performed for all of the 134 key genes as well as for the down-regulated or up-regulated genes respectively, using DAVID; 6.8 beta [50,51]. As shown in Table 2, Cluster 1 (enrichment score 3.47) comprised genes associated with hydrolase function and lipid metabolism, followed by Cluster 2 (enrichment score 3.28) containing lysosome associated genes and Cluster 3 (enrichment score 2.48) containing genes related to saposin-like proteins. Annotation Cluster 4 (enrichment score 2.37) was predominantly composed of cysteine peptidase associated genes. Further Clusters were not considered as they did not contain GO terms of a *p*-value < 0.05. However, 21 out of the 134 genes were associated with the UniProt Keyword “Membrane/Transmembrane” and GO term “integral component of membrane”.

**Table 2:** Functional annotation clusters including up- and down-regulated genes obtained by DAVID.

**Annotation Cluster 1**	**Enrichment Score: 3.47**			
Category	Term	Count	PValue	Benjamini
UP_KEYWORDS	Hydrolase	15	1.3E-4	3.3E-3
GOTERM_MF_DIRECT	hydrolase activity	15	2.3E-4	1.7E-2
GOTERM_BP_DIRECT	lipid metabolic process	7	1.3E-3	3.5E-2
**Annotation Cluster 2**	**Enrichment Score: 3.28**			
Category	Term	Count	PValue	Benjamini
GOTERM_CC_DIRECT	lysosome	6	6.4E-6	1.2E-4
KEGG_PATHWAY	Lysosome	5	2.4E-3	6.7E-3
UP_SEQ_FEATURE	signal peptide	4	9.6E-2	9.1E-1
**Annotation Cluster 3**	**Enrichment Score: 2.48**			
Category	Term	Count	PValue	Benjamini
INTERPRO	Saposin B	4	5.6E-4	3.6E-2
INTERPRO	Saposin-like	4	6.1E-4	2.7E-2
SMART	SapB	3	4.0E-3	3.7E-2
UP_KEYWORDS	Disulfide bond	7	9.1E-2	7.0E-1
**Annotation Cluster 4**	**Enrichment Score: 2.37**			
Category	Term	Count	PValue	Benjamini
GOTERM_CC_DIRECT	lysosome	6	6.4E-6	1.2E-4
SMART	Pept_C1	4	1.5E-4	2.8E-3
INTERPRO	Peptidase C1A, papain C-terminal	4	5.6E-4	3.6E-2
INTERPRO	Peptidase C1A, papain	4	5.6E-4	3.6E-2
GOTERM_MF_DIRECT	cysteine-type endopeptidase activity	4	1.8E-3	4.4E-2
INTERPRO	Cysteine peptidase, histidine active site	3	2.7E-3	6.9E-2
GOTERM_BP_DIRECT	proteolysis involved in cellular protein catabolic process	4	4.5E-3	8.7E-2
INTERPRO	Cysteine peptidase, cysteine active site	3	5.0E-3	1.0E-1
GOTERM_MF_DIRECT	cysteine-type peptidase activity	4	5.8E-3	1.0E-1
GOTERM_CC_DIRECT	extracellular space	5	1.4E-2	8.3E-2
GOTERM_BP_DIRECT	proteolysis	4	3.6E-1	9.5E-1
UP_KEYWORDS	Protease	3	3.7E-1	9.8E-1
GOTERM_MF_DIRECT	peptidase activity	3	4.0E-1	9.9E-1
**Annotation Cluster 5**	**Enrichment Score: 1.20**			
Category	Term	Count	PValue	Benjamini
INTERPRO	Glycoside hydrolase, catalytic domain	3	5.7E-2	6.2E-1
GOTERM_BP_DIRECT	carbohydrate metabolic process	4	6.2E-2	4.8E-1
INTERPRO	Glycoside hydrolase, superfamily	3	6.9E-2	6.1E-1
**Annotation Cluster 6**	**Enrichment Score: 1.06**			
Category	Term	Count	PValue	Benjamini
GOTERM_CC_DIRECT	intracellular membrane-bounded organelle	4	8.3E-3	7.6E-2
GOTERM_MF_DIRECT	glucuronosyltransferase activity	3	6.2E-2	6.1E-1
GOTERM_BP_DIRECT	flavonoid biosynthetic process	3	6.3E-2	4.4E-1
GOTERM_BP_DIRECT	flavonoid glucuronidation	3	6.3E-2	4.4E-1
INTERPRO	UDP-glucuronosyl/UDP-glucosyltransferase	3	6.6E-2	6.3E-1
GOTERM_MF_DIRECT	transferase activity, transferring hexosyl groups	3	9.1E-2	6.9E-1
UP_KEYWORDS	Transferase	7	4.7E-1	9.9E-1
GOTERM_MF_DIRECT	transferase activity	6	5.9E-1	1.0E0
**Annotation Cluster 7**	**Enrichment Score: 0.64**			
Category	Term	Count	PValue	Benjamini
GOTERM_BP_DIRECT	oxidation-reduction process	6	1.8E-1	8.0E-1
UP_KEYWORDS	Oxidoreductase	4	2.6E-1	9.5E-1
GOTERM_MF_DIRECT	oxidoreductase activity	5	2.6E-1	9.6E-1
**Annotation Cluster 8**	**Enrichment Score: 0.53**			
Category	Term	Count	PValue	Benjamini
UP_SEQ_FEATURE	signal peptide	4	9.6E-2	9.1E-1
UP_SEQ_FEATURE	glycosylation site:N-linked (GlcNAc. . .)	3	3.3E-1	9.6E-1
UP_KEYWORDS	Glycoprotein	3	8.2E-1	1.0E0
**Annotation Cluster 9**	**Enrichment Score: 0.18**			
Category	Term	Count	PValue	Benjamini
KEGG_PATHWAY	Metabolic pathways	5	3.3E-1	9.8E-1
UP_KEYWORDS	Zinc	5	6.4E-1	1.0E0
GOTERM_MF_DIRECT	zinc ion binding	5	7.3E-1	1.0E0
UP_KEYWORDS	Metal-binding	6	8.5E-1	1.0E0
GOTERM_MF_DIRECT	metal ion binding	7	9.1E-1	1.0E0
**Annotation Cluster 10**	**Enrichment Score: 0.04**			
Category	Term	Count	PValue	Benjamini
GOTERM_CC_DIRECT	integral component of membrane	21	7.6E-1	9.9E-1
GOTERM_CC_DIRECT	membrane	20	8.7E-1	1.0E0
UP_KEYWORDS	Transmembrane helix	21	1.0E0	1.0E0
UP_KEYWORDS	Transmembrane	21	1.0E0	1.0E0
UP_KEYWORDS	Membrane	21	1.0E0	1.0E0

Interestingly, regarding the non-clustered functional annotation (S3 Table), 53 of the 134 genes were related to the UniProt keyword “Signal” (*p*-value 1.2*10^−9^) suggesting them to be either secreted proteins or part of a membrane. The number of genes associated with this keyword was more or less equally distributed between the up- and down-regulated genes (20 vs. 33 genes, respectively). Further enriched terms mainly included the innate immune response, hydrolases, lipid metabolism, unspecified “metabolic processes” and the lysosome.

Several genes out of the above mentioned clusters (*cpr-3*; *spp-1*, *spp-2*, *spp-3* and *ugt-44* respectively) were additionally identified to be part of the worm’s innate immune response among other differentially expressed genes, such as C-type lectins (*clec-166*, *clec-4*), the caenacin *cnc-11*, the infection response gene *irg-2*, lysozyme *lys-2*, a glutathione-S-transferase (*gst-24*), invertebrate lysozyme *ilys-2*, and *dod-17* (“Downstream Of DAF-16”).

Separate annotation clustering of the down-regulated genes led to an enrichment of very similar terms as obtained for the complete set of differentially regulated genes (S4 Table). Again, genes were related to the lysosome (Cluster 1, ES: 4.95), saposin-like proteins (Cluster 2, ES: 3.29) and cysteine peptidases (Cluster 3, ES: 2.12). Also, the InterPro terms “glycoside hydrolysis” as well as “UDP-glucuronosyl/UDPglucosyltransferase” were significantly enriched. Among the down-regulated genes with the highest fold-changes three “proteins up-regulated in Daf-2” (*pud-1*.*1*, *pud 1*.*2* and *pud-3*) were pinpointed. These *pud* proteins seem to be specific to *Caenorhabditis* as there are no reports of potential homologues in other species. Due to their association with *daf-2* they have been suspected to be somehow involved in aging and lifespan associated processes, but despite intense functional investigations their exact role remains unclear [59].

Regarding the up-regulated genes, no specific function or process was significantly enriched, however, 11 out of 58 genes in total were related to the GO term “integral component of membrane” or the UniProt keywords “Membrane” / “Transmembrane”. Among the genes showing the highest up-regulation were: T22D1.2, an orthologue of human PRB2 and PRB3 (proline-rich protein subfamilies 2 and 3), F13E9.4, which shares 55.7% homology with human filaggrin-2, and Y46H3D.8, homologue (88%) to “human keratin associated protein 9–1” (wormbase.org). Further, *clx-1* (“collagen sequence x–hybridizing”), *numr-1* (“nuclear localized metal responsive”) and several heat shock proteins (*hsp-16*.*1*, *hsp-16*.*48* and *hsp-16*.*11*) that are indicative of a general stress response. Also, homologues to human keratins (K01A6.8; F13E9.14, C28C12.4) and dermokine (H12D21.6) were detected, although their function in *C*. *elegans* remains unclear.

Up-regulated genes also seemed to be involved in the immune response and in defense mechanisms to certain toxins, such as heavy metals, drugs or other chemicals. Interestingly, some of the up-regulated genes, e.g. Y46H3D.8, F13E9.4, *numr-1*, *clx-1*, C28C12.4 and T22D1.2, had previously been found to be differentially regulated upon treatment with hydrolyzable tannins ([60], wormbase.org).

As the number of differentially regulated genes increased with the concentration of the test solutions (Fig 1), the 347 genes that were exclusively up-regulated in the samples treated with 2 mg/mL of fraction H3 were again cross-checked *via* WormBase and annotation data to reveal possible functions affected by the tannin treatment that might have not been detected within the groups’ intersection of 134 genes. However, beside some hints pointing towards a general stress response, nothing could be rationally linked to an activity specific for OPCs.

On the other hand, some of the hypothetical proteins corresponding to genes with increased expression, e.g. C50F7.5, *clx-1* and T22D1.2, were found to be strikingly rich in repetitive sequences containing proline (Table 3). The most impressive example was that of T22D1.2 for which BLASTp analysis revealed a 91% similarity to the human basic salivary proline-rich protein 2 (PRB2) (wormbase.org). The protein consists of a signal peptide (56) followed by repetitive proline sequences and asparagin–alanine–serine–(NAS) triplets possibly indicating N-glycosylation sites [61].

**Table 3:** Predicted protein sequences of T22D1.2, *clx-1* and C50F7.5.

**T22D1.2**	
MRTFQLTLLFTALAVTSLAAPRFSVGETAS*RR****PPPPP****KGTGT****PPPPP****TGE****P****QDLSGEG****NA***	60
***S***RR**PPPPP**KGTGT**PPPPP**TGE**P**QDLSAEEG**NAS**RR**PPPPP**KGTGT**PPPPP**TGE**P**QDLSGE	120
G**NAS**RR**PPPPP**KGTGS**PPPPP**TGE**P**QDLSGEG**NAS**RR**PPPPP**KGTGS**PPPPP**TGE**P**QDLS	240
TEG**NAS**RR**PPPPP**KGTGT**PPPPP**TGE**P**QDLSAEGYASRR**PPPPP**KGTGS**P**T**PPP**TGE**P**QD	300
LSGEG**NAS**RR**PPPPP**KGTGS**PPPPP**TGE**P**QDLSGEG**NAS**RR**PPPPP**KGTGT**PPPP**TGE**P**E	360
KI	362
**CLX-1**	
MFRKALGVLVLVLVAHAVD**P**SDLPD**P**SS**PP**PA**P**R**P**SGQ**PP**G**P**QG**P**SDL**P**G**P**SGA**PP**G**PP**H	60
**P**SG**PP**HR**P**H**P**H**P**SRR**P**R**P**TRL**P**R**P**SRS**P**HSDA**P**E**P**SAATDGFELVFGKHHSTGA**PP**SGG**P**	120
**P**G**P***FD****P****SGA****PP****SGG****PP****GLFD****P****SGA****PP****SGG****PP****G****P****FG****P****SGA****PP****SGG****PP****G****P***FN**P**SEA**PP**SGG**P**	180
TG**P**FD**P**SGA**PP**SGG**PP**G**P**FN**P**SGA**PP**SGG**PP**G**P**FD**P**SGA**PP**SGG**PP**G**P**FN**P**SGA**PP**SGG**P**	240
**P**G**P**FD**P**SGA**PP**SGG**PP**G**P**FD**P**SGA**PP**SGG**PP**G**P**FD**P**SGAQ**P**SGG**PP**G**P**FN**P**SGA**PP**SGG**P**	300
**P**G**P**FD**P**SGA**PP**SGG**PP**G**P**FN**P**SGA**PP**SGG**PP**G**P**FD**P**SGA**PP**SGG**PP**G**P**FD**P**SGA**PP**SGG**P**	360
**P**G**P**FD**P**SGA**PP**SGG**PP**G**P**FN**P**SGA**PP**SGG**PP**G**P**FD**P**SGA**PP**SGG**PP**G**P**FD**P**SGA**PP**SGG**P**	420
**P**G**P**FN**P**SGA**PP**SGG**PP**G**P**FN**P**SGA**PP**SGG**PP**G**P**FD**P**SGA**PP**SGG**PP**G**P**FN**P**SGA**PP**SGG**P**	480
**P**G**P**FD**P**SGA**PP**SGM**PP**V**P**L**P**TDL**P**I**P**SES**P**SFFQWIFGR**P**K**P**SG**P**AG**P**A**P**SGE**PP**G**P**FDP	540
SG**PPP**SESSEGSGI**PP**SF	558
**C50F7.5**	
MQISLTILLAVAGATFAA**P**SDLGRGHHHHHHHHHKTKA**P**RTSRGIATTTFA**P**TSSDL**P**IA	60
GSSSA**P**VIASSAD**P**IL**P**TSVV**P**Q**P**SNE**P**S**P**GTVA**P**SDE**P**S**P**SG**PP**S**P**G**P**VN**P**SED**P**Q**P**SG	120
**PP**S**P**G**P**VD**P**SED**P**Q**P**SVE**P**SEDHQ**P**SG**PP**S**P**G**P**VD**P**SED**P**Q**P**SVE**P**SED**P**Q**P**SG**PP**S**P**G**P**	180
VD**P**SED**P**Q**P**SGSSS**P**G**P**VD**P**SDE**P**S**P**SG**PP**S**P**G**P**VD**P**SED**P**K**P**SE**PP**S**P**G**P**VD**P**SDE**P**S**P**	240
SD**PP**G**PP**G**PP**G**PP**TRR**PP**G**PP**G**PP**TRR**PP**G**PP**G**PP**TRR**PP**G**PP**G**PP**HHHDHGHHGHHGHH	300
FDQEQL	306

P: Proline, NAS: Potential N-glycosylation site. Repetitive sequences are displayed in italics and the complete site of repetition is underlined.

Seven genes (Y17D7B.10, *tag-38*, F35E8.19, F08A7.1, T16H12.9, *ttr-36* and Y106G6D.8) seemed to be differentially regulated exclusively in the group treated with crude extract “RE”. However, this was most likely due to the cut-off set at a fold-change of 2.0 and not to additional effects caused by other components of the extract than procyanidins, as the values for groups “H3” were slightly below and those for “RE” were slightly above the threshold of 2.0.

### Quantitative RT-PCR

**Fig 2:**
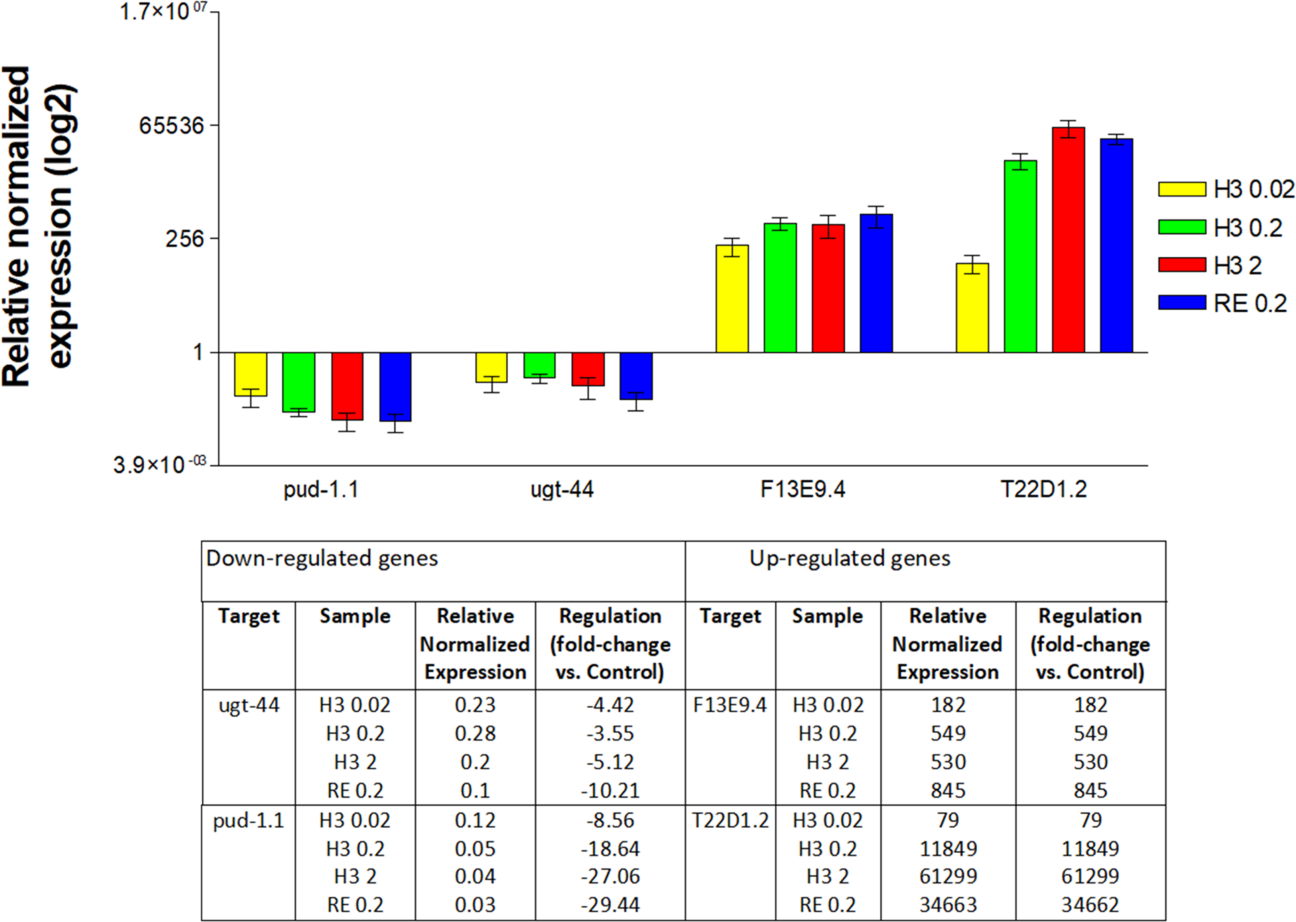
Quantitative RT-PCR. Relative normalized expression of *ugt-44*, *pud-1*.*1*, F13E9.4 and T22D1.2 mRNA following the treatment with OPC enriched fraction “H3” or *C*. *mucronatum* extract (ethanol: water 1:1; “RE”); 0.02, 0.2 and 2: concentrations of 0.02 mg/mL, 0.2 mg/mL and 2 mg/mL respectively. Data are presented on a log2 scale, the line at 1.0 indicates the expression level of the negative control (DMSO 1%).

To confirm and cross-validate the results of the microarray, qPCR was performed to monitor the expression of selected genes in three independent experiments, receiving the same treatment as the samples prepared for the microarray. F13E9.4 and T22D1.2 were selected as representatives among the up-regulated genes, *pud-1*.*1* and *ugt-44* were chosen from the down-regulated genes (for primer sequences see Materials and Methods). Normalized levels of mRNA expression for each gene are shown in Fig 2. In general, the results obtained by qPCR support the findings of the microarray experiment. A clear concentration-dependent regulation was observed for *pud-1*.*1* (down) and T22D1.2 (up) in the groups treated with fraction H3. Again, the increase in the expression of T22D1.2 was extraordinarily strong during qPCR analysis. The expression of *ugt-44* was almost exactly in the same range for all treated groups as in the array. F13E9.4 was rather strongly up-regulated in both experiments, but markedly below the expression of T22D1.2.

Similar to the array results, the differential expression in the groups treated with the crude extract (0.2 mg/mL) exceeded that of the groups treated with H3 at the same concentration, and for *pud-1*.*1*, *ugt-44* and F13E9.4 even the groups treated at 2 mg/mL.

### GFP reporter gene constructs

Since the function of T22D1.2 has not been described so far, its site of expression and secretion was attempted to be revealed by reporter gene constructs using the gene promoter fused to GFP. In order to localize the secretion site, worms containing a GFP construct of the promoter sequence followed by the signal peptide, were treated with the hydro-ethanolic extract at two concentrations (0.2 mg/mL and 2 mg/mL). Unfortunately, the GFP signal in the treated samples was extremely low and hardly detectable (S1 Fig). Possibly, the gene was expressed at a very low rate, somehow contradicting the array results, or the low fluorescence was caused by secretion of GFP into the intestine due to the signal peptide, followed by quick degradation by proteases.

**Fig 3:**
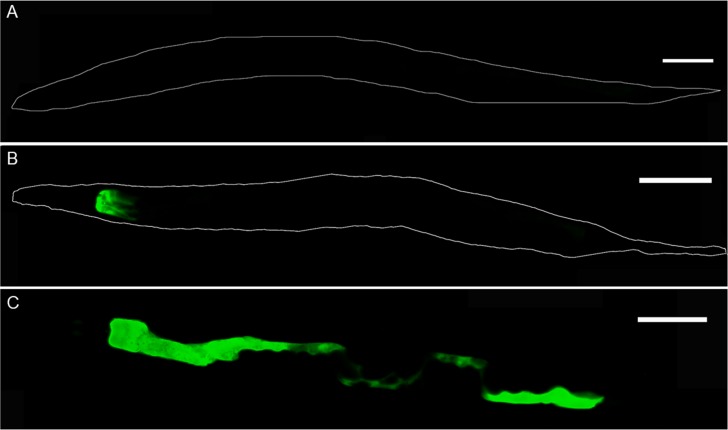
Representative images of *C*. *elegans* expressing pT22D1.2::GFP depending on treatment with hydroethanolic extract from *C*. *mucronatum*. A: negative control (DMSO 1%); B: treatment with 0.2 mg/mL extract; C: treatment with 2 mg/mL extract. Scale bars = 100 μm.

Therefore, the promotor sequence was subsequently fused to GFP without the signal peptide. In this case a strong, concentration dependent GFP signal was observed particularly in the group treated with 2 mg/mL of the extract, but also at 0.2 mg/mL (Fig 3). The main expression was observed in the intestine, particularly the part posterior to the pharynx, extending to the entire intestine with increasing concentration. No expression at all was seen in the negative controls.

**Fig 4:**
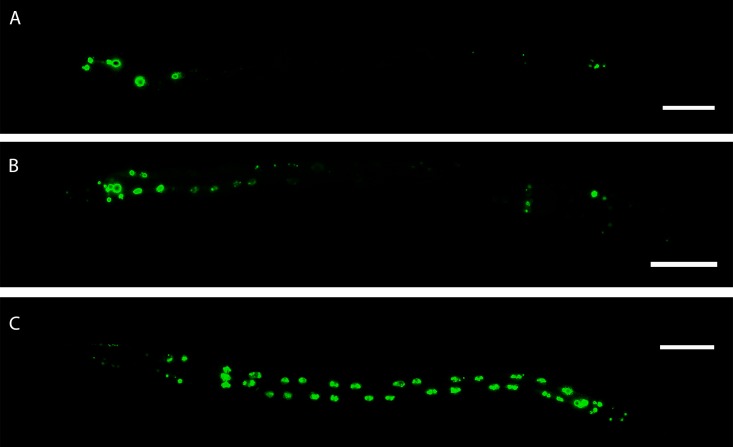
Representative images of *C*. *elegans* expressing *numr-1*::GFP depending on treatment with the hydroethanolic extract from *C*. *mucronatum*. A: negative control (DMSO 1%); B: treatment with 0.2 mg/mL extract; C: treatment with 2 mg/mL extract. Scale bars = 100 μm.

*Numr-1*, which was found to be strongly increased by the procyanidins during the transcriptome analysis, has previously been reported as an indicator exclusively for metal induced stress [62]. A *C*. *elegans* strain containing a *numr-1* promoter::GFP construct was treated with the extract, in order to assess whether the transcription could also be induced by stressors other than metals. As displayed in Fig 4, basal gene expression was mainly observed in tail and head, but in some cases also in the intestine of the negative controls. Treatment with the extract at a concentration of 0.2 mg/mL slightly increased the expression of *numr*-1 in head and tail compared to the negative control whereas a concentration of 2 mg/mL led to a strong fluorescence of the nuclei throughout the worms’ body, mainly in the intestine.

In summary, both genes, T22D1.2 and *numr-1* showed a concentration-dependent increase in the expression as well as an extension of the expression site after contact of the worm with oligomeric procyanidins, visually confirming the microarray results. Taken together, it can be proposed that the main site of action of proanthocyanidins is the worms’ intestine.

## Discussion

Condensed tannins are known for their ability to interact with certain proteins mainly *via* hydrogen bonding [19,21,22], therefore, it is very likely that the observed differential gene expression is based on an interaction of the proanthocyanidins with proteins of the nematode in a more or less specific way. Particularly proline- or histidine-rich proteins are suspected to be possible interaction partners due to the high affinity of tannins in general to these proteins [20,25,63].

As previous microscopic studies revealed structural alterations in the cuticle of different parasitic nematodes [14,29] and *C*. *elegans* [26], tannins have been suspected to bind to sheath or cuticle which are composed of collagen-like and non-collagen-like proteins (cuticlins) that are both rich in proline and hydroxyproline [64,65]. Also, the hypodermis, that seemed to be detached from the muscle tissue below, the muscle tissue itself and particularly the intestine which showed severe signs of damage and digestion, were strongly affected by tannin treatment [28,31,33,34,66].

The results from the current study suggest that an alteration of gene expression most frequently occurs for intestinal proteins and enzymes as the intestine is the tissue mainly exposed to tannins. However, this finding could also be due to the short incubation time with the substances and other tissues might also be affected following a longer exposure.

A common feature of many differentially regulated genes within this study is their localization to membranes. However, regarding the sequences of the respective corresponding proteins, none of these proteins “integral to membrane” is particularly enriched in either proline or histidine and might therefore not act as a direct binding partner of PAC. More likely, PACs exert membrane destabilizing effects that could then affect membrane-bound enzymes or transporters, similar to the activity recently described for a trimeric A-type procyanidin against *Bacillus cereus* [67]. Moreover, flavonoids and tannins are also capable of chelating a variety of polyvalent metal cations via their o-dihydroxy function in the B-ring or involving the 4-oxo function of flavones [68–72] and the chelating activity seems to increase with the molecular size of condensed tannins [72]. Therefore, an interference of the tannins (and flavonoids) with certain ions within the worm (e.g. iron or zinc as part of metalloproteases), similar to previously described deleterious effects in mammals (reviewed by [63]) and bacteria [67,73], is also feasible.

The (mainly down-regulated) enzymes clustered according to their structure or functions as hydrolases, part of the lysosome, saposin-like proteins, cysteine peptidases or proteins associated with the worms’ immune response, also share the feature of possessing a signal peptide, indicating their extracellular location. Therefore, these enzymes are possibly affected by the treatment due to their accessibility for PAC and general detrimental effects on membranes rather than due to their specific function or structure. In line with this hypothesis is the absence of any receptor mediated response or of specific signal pathways activated or inactivated by the treatment.

Finally, three members of the hitherto uncharacterized PUD family (proteins up-regulated in daf-2), *pud-1*.*1*, *pud-1*.*2* and *pud-3* were among the strongest down-regulated genes. Due to their association with *daf-2* they have been suspected to be somehow involved in aging and lifespan associated processes and were suggested to act as transcription regulators for collagen genes, but despite intense functional investigations their exact role remains unclear [59].

T22D1.2 was the gene that was by far up-regulated the strongest with a fold-change > 900 at the highest concentration of H3. As mentioned above, it is an orthologue of human basic salivary proline-rich protein 2, however, not much is known about its function in *C*. *elegans*, except for one knockdown study and subsequent treatment with mercury chloride revealing a phenotype hypersensitive to chemicals [74]. As shown in Fig 3, the intestine is the main expression site and the expression increases with the amount of test substance. Following translation of T22D1.2 in the enterocytes, the protein enters the secretory pathway as indicated by the presence of a signal peptide [56]. Unfortunately, the final localization of the protein could not be unambiguously determined as the construct containing the signal peptide only led to an extremely weak fluorescence (S1 Fig). Possibly, the protein was immediately degraded after secretion to the intestinal lumen, the fluorescence signal has been quenched or the protein was rapidly excreted due to the very frequent defecation of the worm [75]. Regarding the role of T22D1.2, it can be seen as a defense mechanism against tannins in the soil-inhabiting *C*. *elegans*, similar to salivary proteins in mammals (reviewed by [76]). Tannins can interact with proteins, calcium and iron absorption or digestive enzymes and lead to growth impairment and weight loss in animals. On the other hand, proline-rich salivary proteins are an effective mechanism in many animals to bind tannins and to prevent these detrimental effects (reviewed by [63,77,78]. In some species, i.e. rats and mice, proline-rich proteins are not constitutively expressed, but are induced by tannins [63,79]. Toxic effects of tannins in invertebrates have frequently been described for different species of plant-herbivore insects. In sensitive species, severe lesions of the midgut have been detected whereas other species remained unaffected (reviewed by [80]. The underlying mechanism is still unclear, a direct effect of tannins binding to epithelial membranes has been discussed as well as an indirect effect by oxidative stress, as insect guts contain iron (Fe^3+)^ as a potential oxidant [80]. However, in cases where oxidative stress was involved in the toxicity against *C*. *elegans*, typical detoxifying enzymes, such as glutathione-S-transferases, should be expected to be up-regulated more pronouncedly [81,82]. Concerning defense mechanisms in insects, the presence of surfactants and possibly hydrolases as well as several ways of inhibiting a possible oxidation of tannins have been described. On the other hand, the presence of tannin-binding proteins, similar to salivary proteins, has been suspected, but has not been identified until now [80]. Possibly, T22D1.2 could provide such a detoxification mechanism in *C*. *elegans*, but further studies are ongoing to confirm the function of this gene and the related protein.

Not only proline-rich salivary proteins, but also histidine-rich proteins, such as histatins can interact with tannins [63,83]. Therefore, also the histidine rich NUMR-1 [62] is potentially involved in the defense as a binding protein. However, as it is localized within the nucleus, a direct interaction with tannins seems very unlikely. *Numr-1* (nuclear localized metal responsive) which showed a strongly increased expression, has also been shown to be strongly up-regulated upon treatment with tannic acid, but not after treatment with quercetin [60]. The corresponding protein NUMR-1 was localized in the nuclei of head neurons, egg-laying muscles of the vulva and cells in the tail [62] and was found to be induced under metal stress, particularly by cadmium and copper. Other stressors such as juglone, tunicamycin, heat shock, starvation or infections with pathogens did not cause an increased transcription [62]. The expression pattern of NUMR-1::GFP induced at 100 μM cadmium [62] resembled that of 2 mg/mL EtOH-H_2_O (1:1) extract from *C*. *mucronatum* (Fig 4), although slightly more nuclei seemed to be fluorescing upon extract treatment. As it is unlikely that the result observed for the extract and the respective fractions obtained from this extract is caused by heavy metals (e.g. as a contamination) and also commercially available purified tannic acid caused an up-regulation of this gene [60], *numr-1* could be part of a more or less specific stress response.

## Conclusion

The current study provides first insights into transcriptomic changes in *C*. *elegans* upon treatment with a proanthocyanidin-enriched extract from *C*. *mucronatum* at different concentrations. Despite the lack of distinct signal pathways or receptors affected, the findings strongly point towards proteins within the intestinal membrane as well as membrane bound and secreted enzymes and peptides to be the main target for PACs. Most likely, the observed alterations in gene expression are caused indirectly *via* detrimental effects of the PACs on membranes, nevertheless, the current results provide an insight into the molecular processes affected by tannin treatment as a potential base for future drug development. Further, T22D1.2 which was massively up-regulated was identified as a possible detoxification mechanism.

## Supporting information

S1 Table Processed data of the microarray experiment. (XLSX)

S2 Table Intersection of differentially expressed genes in the treated samples versus the negative control showing at least a 2-fold difference and a p-value < 0.05. H3 0.02 / 0.2 / 2: samples treated with fraction H3 in concentrations of 0.02, 0.2 or 2 mg/mL resp.; RE 0.2: samples treated with 0.2 mg/mL crude extract.
(XLSX)

S3 Table Non-clustered functional annotation table of the differentially regulated genes among all treated groups. (XLSX)

S4 Table Annotation Clusters of the down-regulated genes among all treated groups versus the negative control. (XLSX)

S1 Fig Representative images of *C. elegans* expressing pT22D1.2::GFP including the signal peptide. A: negative control (DMSO 1%); B: treatment with 2 mg/mL extract. Scale bars = 100 μm.
(DOCX)

## Abbreviations

ES: enrichment score
LC: lethal concentration
NGM: nematode growth medium
qPCR: quantitative real time PCR
OPC: oligomeric procyanidins
PAC: proanthocyanidins
H3: OPC enriched fraction “H3”
RE: crude extract (ethanol: water 1:1)
SP: signal peptide
