# Supplementary material for: Transcriptome analysis reveals molecular anthelmintic effects of procyanidins in C. elegans
Source: PLoS One. 2017 Sep 19;12(9):e0184656. doi: 10.1371/journal.pone.0184656 (PMC5604969; doi:10.1371/journal.pone.0184656)

**S1 Fig: Representative images of *C. elegans* expressing pT22D1.2::GFP including the signal peptide.** A: negative control (DMSO 1 %); B: treatment with 2 mg/mL extract. Scale bars = 100 µm.


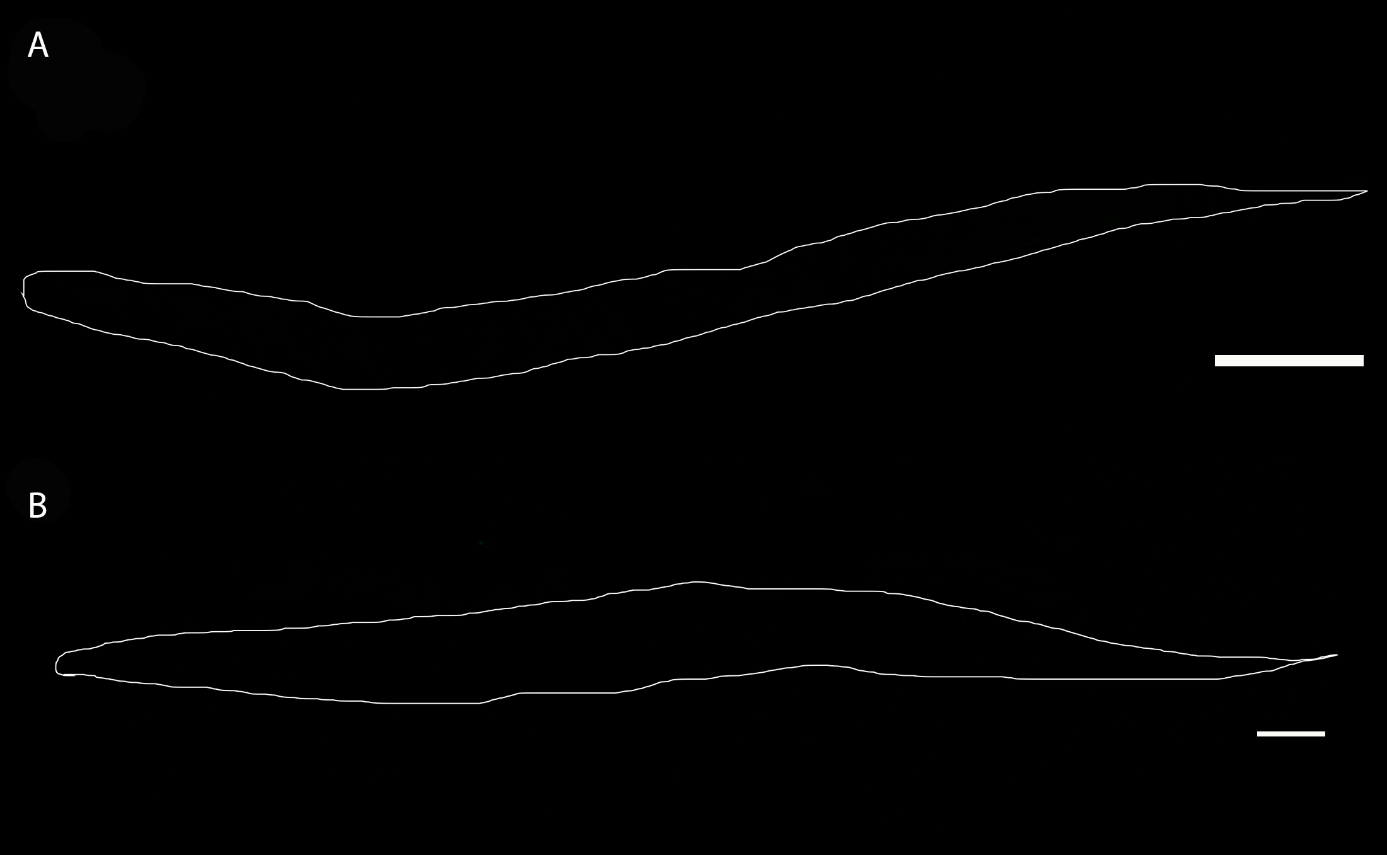

Supplement: S1 Fig — A: negative control (DMSO 1%); B: treatment with 2 mg/mL extract. Scale bars = 100 μm. (DOCX) [file pone.0184656.s005.docx]
